# Sugar uptake by the solventogenic clostridia

**DOI:** 10.1007/s11274-015-1981-4

**Published:** 2016-01-09

**Authors:** Wilfrid J. Mitchell

**Affiliations:** School of Life Sciences, Heriot-Watt University, Riccarton, Edinburgh, EH14 4AS UK

**Keywords:** ABE fermentation, Sugar uptake, Phosphotransferase system, Catabolite repression

## Abstract

The acetone–butanol–ethanol fermentation of solventogenic clostridia was operated as a successful, worldwide industrial process during the first half of the twentieth century, but went into decline for economic reasons. The recent resurgence in interest in the fermentation has been due principally to the recognised potential of butanol as a biofuel, and development of reliable molecular tools has encouraged realistic prospects of bacterial strains being engineered to optimise fermentation performance. In order to minimise costs, emphasis is being placed on waste feedstock streams containing a range of fermentable carbohydrates. It is therefore important to develop a detailed understanding of the mechanisms of carbohydrate uptake so that effective engineering strategies can be identified. This review surveys present knowledge of sugar uptake and its control in solventogenic clostridia. The major mechanism of sugar uptake is the PEP-dependent phosphotransferase system (PTS), which both transports and phosphorylates its sugar substrates and plays a central role in metabolic regulation. Clostridial genome sequences have indicated the presence of numerous phosphotransferase systems for uptake of hexose sugars, hexose derivatives and disaccharides. On the other hand, uptake of sugars such as pentoses occurs via non-PTS mechanisms. Progress in characterization of clostridial sugar transporters and manipulation of control mechanisms to optimise sugar fermentation is described.

## Introduction

Two of the most important issues facing the world at the present time are energy security and climate change. For over 100 years, the most important source of energy has been burning of fossil fuels, releasing CO_2_ which as a greenhouse gas is contributing to global warming. Furthermore, around the globe, there is a widespread recognition that continued reliance on a finite energy source is unsustainable, resulting in increased emphasis on development of renewable and environmentally friendly alternatives. Biofuels produced by microbial fermentations are viewed as a viable option, with bioethanol and biodiesel already in widespread use as transportation fuel extenders. A more attractive biofuel is biobutanol, which is produced by the solventogenic clostridia in the acetone–butanol–ethanol (ABE) fermentation (Schiel-Bengelsdorf et al. 2013). The ABE fermentation was introduced at the time of the First World War to produce acetone required for cordite manufacture, and subsequently the by-product butanol became important as a solvent in production of nitrocellulose lacquer sprays for the expanding automobile industry. However, by the second half of the twentieth century the ABE fermentation in most developed countries had succumbed to economic competition from petrochemical-based production. Nevertheless, the considerable importance of butanol as a bulk industrial chemical, coupled with its advantageous properties as a biofuel, has stimulated interest in re-establishment of the fermentation process (Dürre 2011; Green 2011).

A key requirement in revival of the ABE fermentation is the engineering of bacterial strains with improved properties and increased productivity, and recently developed methods for genetic manipulation of the clostridia now offer encouragement that this is a feasible goal. Thus, various metabolic engineering strategies have been applied to improvement of butanol tolerance and alteration of end-product formation (Lütke-Eversloh 2014). Nevertheless, since the major contributor to the operational costs of the ABE fermentation is the substrate, an economic process will necessarily be dependent on the use of cheap feedstocks, probably obtained as waste materials and containing a range of fermentable carbohydrates (Gu et al. 2014). Therefore, given the propensity of bacteria to regulate uptake of sugars in response to the nutritional status of their environment, an understanding of the mechanisms of sugar uptake and its control in clostridia is essential in order to enable metabolic engineering strategies to be designed to ensure optimum substrate utilization and fermentation performance. This review will summarise current understanding of sugar transport mechanisms, and progress in manipulation of associated regulatory systems in the solventogenic, butanol-forming clostridia.

## Sugar uptake by the phosphotransferase system

**Fig. 1:**
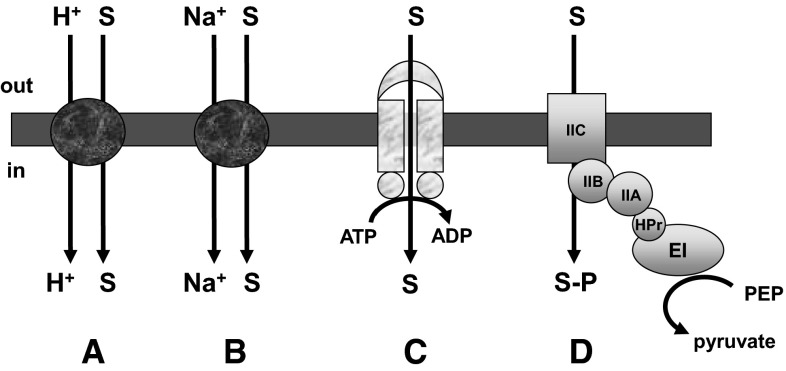
Bacterial sugar transport mechanisms. **A** H^+^-symport, in which the uptake of sugar occurs concurrently with a proton, driven by a transmembrane proton gradient, **B** Na^+^-symport, in which the uptake of sugar occurs concurrently with a sodium ion, driven by a transmembrane sodium gradient, **C** ABC (ATP-binding cassette) system supporting sugar uptake via ATP hydrolysis, **D** PEP-dependent phosphotransferase system coupling sugar uptake to phosphorylation. S = sugar

Bacteria utilise a small number of mechanisms to support the uptake of sugars and other nutrients. With the exception of facilitated diffusion which is found relatively rarely, energy is coupled to the trans-membrane transport process in order to support accumulation of the substrate, and transport systems are classified on the basis of the energy coupling mechanism (Fig. 1). In common with other anaerobes, the clostridia show a marked dependence on the PEP-dependent phosphotransferase system (PTS), which unlike other transport mechanisms catalyses both the accumulation and chemical conversion (phosphorylation) of its carbohydrate substrates. The central feature of the PTS is a multicomponent phosphoryl transfer chain comprising two general proteins, enzyme I (EI) and HPr, and two system-specific proteins or domains referred to as IIA and IIB. A final system-specific protein/domain IIC (in some cases together with an additional protein/domain IID) provides the channel by which the sugar crosses the membrane to be phosphorylated as it enters the cytoplasm. The PEP-dependent phosphorylation of substrate can be followed in permeabilised cells or cell free extracts, and the demonstration of this activity provides evidence for a functional PTS. By this criterion, a range of phosphotransferase activities has been demonstrated in the butanol-forming clostridia, indicating the importance of the PTS in uptake of sugars and sugar derivatives in these strains (Table 1).

**Table 1:** Phosphotransferase systems in solventogenic clostridia

Strain	Substrates^b^	Number of complete phosphotransferases encoded by genome^d^	References
*C. acetobutylicum* ATCC 824	Cellobiose, fructose, galactose, glucose, lactose, maltose and sucrose	13	Tangney and Mitchell (2000, 2007), Tangney et al. (2001), Mitchell and Tangney (2005), and Yu et al. (2007)
*C. acetobutylicum* DSM 792 (=ATCC824)	Mannitol	13	Behrens et al. (2001)
*C. acetobutylicum* ATCC 4259 (=DSM1731)	Glucose	14	Hutkins and Kashket (1986)
*C. beijerinckii* NCIMB 8052 (formerly *C. acetobutylicum*)^a^	Fructose, glucitol, glucose, lactose, mannitol, *N*-acetylglucosamine and sucrose	43	Mitchell et al. (1991), Mitchell (1996), Tangney et al. (1998a,b), Lee et al. (2001) and Al Makishah and Mitchell (2013)
*C. saccharobutylicum* NCP262 (=DSM13864) (formerly *C. acetobutylicum*)^a^	Galactose, glucose and lactose	14	Gutierrez and Maddox (1996) and Diez-Gonzalez and Russell (1996)
*C. saccharoperbutylacetonicum* N1-4 (HTM) (=DSM 14923) (formerly *C. acetobutylicum*)^a^	nd^c^	31	
*C. pasteurianum* ATCC 6013	Fructose, glucitol, glucose, mannitol and sucrose	14	Hugo and Gottschalk (1974) and Booth and Morris (1982)
*C. pasteurianum* NCIMB 9486 (=ATCC 6013)	Glucitol and glucose	14	Mitchell and Booth (1984) and Roohi and Mitchell (1987)

^a^Strains re-classified in 2001 (Keis et al. 2001)

^b^PTS substrates confirmed by activity assays

^c^None determined

^d^Genomes searched at KEGG Genomes (http://www.genome.jp/kegg/catalog/org_list.html); genome sequences are also available at NCBI (http://www.ncbi.nlm.nih.gov/genomes/MICROBES/microbial_taxtree.html). A complete phosphotransferase system is defined as having a minimum of IIB and IIC domains; some phosphotransferase systems are known to lack a IIA domain but may obtain this function by interacting with a component of an alternative system (Mitchell and Tangney 2005)

The significance of the PTS in sugar uptake by *Clostridium acetobutylicum* and *Clostridium beijerinckii* has been further substantiated by genome sequencing which has indicated the capacity to encode 13 and 43 complete systems, respectively (Table 1; Mitchell 2015). These phosphotransferases are related to those in other bacteria, and exhibit similar organizational characteristics. For example, genes encoding IIA, IIB and IIC domains (and a IID domain where present) are found in operons which often also encompass genes specifying enzymes involved in the metabolism of phosphorylated PTS products, thus allowing for coordinated control of sugar uptake and metabolism (Tangney and Mitchell 2005; Mitchell 2015); domains can be present in different orders within a single protein or found in different combinations on separate proteins (Mitchell and Tangney 2005; Mitchell 2015); some genes or operons do not encode a IIA domain, suggesting that the IIA function of the phosphotransferase is provided by another system (Reid et al. 1999; Tangney et al. 2001; Mitchell 2015). On the other hand, an unusual feature of the clostridial PTS is that the *ptsI* (enzyme I) and *ptsH* (HPr) genes are not contiguous, perhaps indicative of as yet uncharacterised features of metabolic regulation, and the encoded proteins form phylogenetic groups distinct from those of other bacteria (Mitchell 2015).

The substrates of the individual PTSs in *C. acetobutylicum* have been deduced largely on the basis of genome context and gene expression studies, and are summarised in recent reviews (Gu et al. 2014; Mitchell 2015). However, verification through biochemical characterization of individual systems has generally not been reported. Physiological analysis correlating gene expression with PTS synthesis and properties identified systems responsible for uptake and phosphorylation of sucrose (Tangney and Mitchell 2000), mannitol (Behrens et al. 2001), maltose (Tangney et al. 2001) and lactose (Yu et al. 2007). These designations were largely supported by a subsequent transcriptional analysis of gene expression in cultures grown on 11 different carbon sources, from which the functions of several of the remaining phosphotransferases could also be inferred (Servinsky et al. 2010). A recent mutational study in which putative fructose phosphotransferases were systematically deleted or inactivated confirmed that the PTS encoded by *cacc0233*/*cac0234* in *C. acetobutylicum* ATCC 824 is a fructose PTS, consistent with the fact that *cac0232* encodes a putative 1-phosphofructokinase (Voigt et al. 2014). The same study implicated the PTS encoded by *cac1457*/*cac1458*/*cac1459*/*cac1460* in mannose transport. In both cases, however, residual uptake of the respective sugars was observed to occur in the mutants. It is therefore apparent that a redundancy of phosphotransferases, or in some cases perhaps other uptake systems, with respect to substrate specificity will limit the extent to which PTS function can be characterised via mutational analysis.

The number of phosphotransferases in *C. beijerinckii* represents a greater challenge with respect to functional characterization, particularly since extensive redundancy of function is inevitable. Nevertheless, expression and mutational studies on *C. beijerinckii* NCIMB 8052 clearly implicated *cbe5012* as encoding a sucrose PTS (Reid et al. 1999). More recently, Al Makishah and Mitchell (2013) cloned the PTS encoded by *cbe4532*/*cbe4533* and showed that it complemented *E. coli* mutants defective in uptake and phosphorylation of glucose and *N*-acetylglucosamine, while a similar approach has demonstrated that the PTS encoded by *cbe0751* can transport glucose and mannose (Essalem and Mitchell, manuscript in preparation). The single member of the glucitol PTS family encoded in the *C. beijerinckii* genome (*cbe0336*/*cbe0337*/*cbe0339*) shows conservation of the unique domain structure associated with the glucitol PTS family, in which the IIC domain is divided between two proteins, one of which also contains the IIB domain. Unsurprisingly, expression of this PTS was correlated with growth on glucitol, thus confirming its function (Tangney et al. 1998a). Beyond these four systems, the functions of phosphotransferases in *C. beijerinckii* have so far only been inferred on the basis of genome context and sequence analysis (Shi et al. 2010; Gu et al. 2014; Mitchell 2015).

Two other species, *Clostridium saccharobutylicum* and *Clostridium saccharoperbutylacetonicum* are included in the solventogenic group (Keis et al. 2001), while *Clostridium pasteurianum* has been shown to produce butanol under certain growth conditions (Mitchell 1998). Sugar uptake in these bacteria has received little experimental attention. There is, however, evidence for the existence of some phosphotransferase activities, and recently completed genome sequences (Del Cerro et al. 2013; Poehlein et al. 2013, 2014, 2015; Rotta et al. 2015) have revealed the presence of multiple phosphotransferase systems, re-confirming the importance of the PTS as a mechanism of sugar uptake in the solventogenic clostridia (Table 1).

## Sugar uptake by non-phosphotransferase mechanisms

By comparison with the PTS, non-PTS mechanisms of nutrient accumulation by the clostridia have received very little attention. Nevertheless, numerous non-PTS transporters that could potentially be involved in sugar uptake are encoded in the genomes of all solventogenic clostridial strains. An early and detailed study carried out on *C. pasteurianum* demonstrated that uptake of galactose and gluconate resulted in alkalinisation of the medium, thus suggesting that the uptake mechanism was via H^+^- symport (Booth and Morris 1975). Further evidence in support of this conclusion was provided in that accumulation of the substrate was sensitive to treatments which collapsed the transmembrane proton gradient, including inhibition of the membrane-bound ATPase complex responsible for generating the gradient (Clarke et al. 1979); also, artificially generated ion gradients could support substrate accumulation, and a putative *pts* mutant showed normal accumulation of galactose and gluconate (Booth and Morris 1975, 1982). Although direct studies of the uptake mechanisms have not been performed, effects of energy inhibitors and/or and lack of significant PEP-dependent phosphorylation in cell extracts has been taken as evidence for the existence of non-PTS-mediated uptake of maltose by *C. beijerinckii* (Albasheri and Mitchell 1995), and of galactose by *C. beijerinckii* (Mitchell 1996) and *C. saccharobutylicum* (Gutierrez and Maddox 1996). In all of the above cases, the demonstration of enzyme activities which will initiate the metabolism of the non-phosphorylated substrates has provided additional credence to the suggestion that non-PTS-mediated routes of assimilation play an important role in their utilization (Bender et al. 1971; Daldal and Applebaum 1985; Albasheri and Mitchell 1995; Mitchell 1996).

Recent interest in non-PTS sugar uptake by the solventogenic clostridia has centred on the pentoses xylose and arabinose. These sugars are principal constituents of lignocellulose, which is viewed as an attractive feedstock for the ABE fermentation (Jurgens et al. 2012). Analysis of clostridial genome sequences has identified genes encoding putative xylose transporters; an ABC transporter in *C. beijerinckii*, and potential proton symporters in both *C. acetobutylicum* and *C. beijerinckii.* Three symporters in *C. acetobutylicum*, encoded by *cac1339*, *cac1345* and *cac3451*, have been shown to be induced under conditions in which xylose is being used as a substrate (Grimmler et al. 2010; Servinsky et al. 2010). Of these only the *cac1345* gene, referred to as *xylT*, has been confirmed to encode a xylose transporter since its disruption resulted in a significant impairment of growth on xylose, its expression complemented a *xylE* (xylose transporter) mutant of *E coli*, and its overexpression resulted in enhanced xylose uptake by *C. acetobutylicum* (Gu et al. 2010; Xiao et al. 2011; Li et al. 2013). In the case of *C. beijerinckii*, similar functional analyses have characterised the *cbe0109* (*xylT*) and *cbe2380*-*2382* (*xylFGH*) gene products as xylose transporters (Xiao et al. 2012; Sun et al. 2015). Since inactivation of neither of these systems resulted in complete loss of growth on xylose, it appears as though they both can contribute to uptake of the sugar. Interestingly, a detailed analysis has shown that the XylFGH ABC transporter is subject to a novel mechanism of induction in response to xylose, which involves a membrane bound sensor related to the XylF protein (Sun et al. 2015).

In the case of arabinose, a gene cluster in *C. acetobutylicum* (*cac1339*–*cac1349*) has been shown to be under control of a putative AraR regulator, and some of the genes in this region including *cac1339* were found to be induced by arabinose as well as by xylose (Servinsky et al. 2010; Zhang et al. 2012). It is therefore possible that the putative transporters encoded by *cac1339* and *cac1345* (*xylT*) are involved in uptake of both pentose sugars, but this has yet to be examined. Furthermore, expression of genes *cac1529* and *cac1530* encoding, respectively, a putative arabinosidase and a symporter protein is also induced by arabinose and is under AraR control (Servinsky et al. 2010; Zhang et al. 2012). A putative ABC transporter (AraFGH, encoded by *cbe4448*–*cbe4450*) has been identified in *C. beijerinckii* (Gu et al. 2014), but as yet there is as no direct evidence for its role in arabinose uptake.

A study of a mutant strain of *C. beijerinckii* has indicated the potential importance of non-PTS uptake of glucose in the ABE fermentation, despite the presence of a glucose PTS in this bacterium. The mutant, BA101, was isolated from the parental strain NCIMB 8052 following chemical mutagenesis and selection for resistance to the glucose analogue 2-deoxyglucose (Annous and Blaschek 1991). As might have been expected the mutant showed a decreased PTS activity for glucose (Lee et al. 2001), but conversely displayed an increased utilization of glucose in culture as well as increased solvent production (Formanek et al. 1997). An elevated level of the enzyme glucokinase in the BA101 strain, which was most pronounced in the solventogenic growth phase, suggested the possibility of an associated non-PTS mechanism of glucose uptake (Lee et al. 2001). Evidence for the presence of such an alternative glucose uptake system was subsequently obtained by comparing the characteristics of glucose uptake and phosphorylation by the parental and mutant strains (Lee et al. 2005). As cultures progressed from the acidogenic (exponential) to solventogenic (stationary) phase, glucose uptake was found to exhibit an increased dependence on the transmembrane proton gradient as shown by effects of energy inhibitors, accompanied by changes in sensitivity to substrate analogues. At the same time, an increase in the proportion of glucose phosphorylated by cell extracts via glucokinase as opposed to the PTS was observed. It thus appeared that the glucose PTS became less influential at later stages of fermentation, during which time the fermentation products accumulated in the culture.

**Fig. 2:**
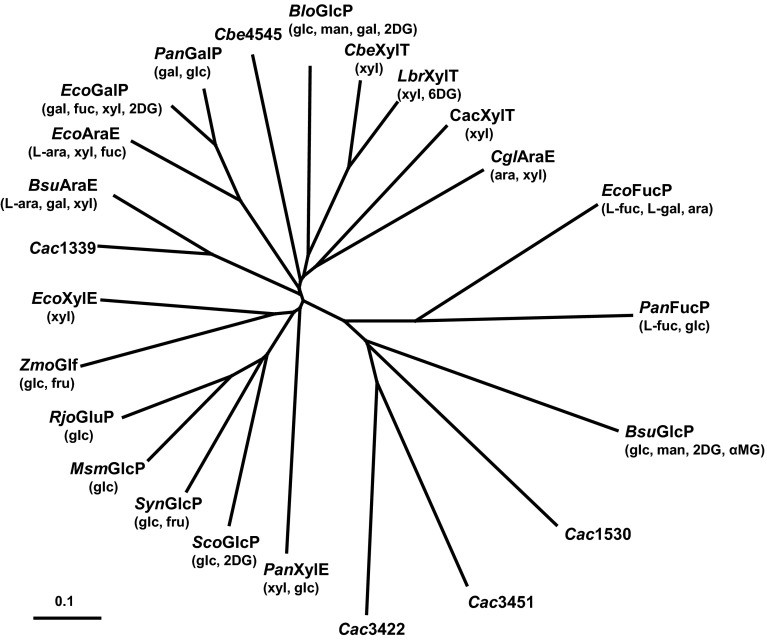
Phylogenetic tree of characterised bacterial hexose and pentose transporters belonging to the major facilitator superfamily. Clostridial protein sequences *Cac*XylT (*Cac*1345), *Cac*1339, *Cac*1530, *Cac*3422 and *Cac*3451 of *C. acetobutylicum* and *Cbe*XylT (*Cbe*0109) and *Cbe*4545 of *C. beijerinckii* were obtained from the *C. acetobutylicum* (http://www.ncbi.nlm.nih.gov/nuccore/15893298) and *C. beijerinckii* (http://www.ncbi.nlm.nih.gov/nuccore/150014892?report=genbank) genome websites. Other sequences included in the analysis are as follows: *Blo*GlcP, *Bifidobacterium longum* GlcP WP_011068757.1 (Parche et al. 2006); *Bsu*AraE, *Bacillus subtilis* AraE WP_003243899.1 (Krispin and Allmansberger 1998); *Bsu*GlcP, *B. subtilis* GlcP WP_003245772.1 (Paulsen et al. 1998); *Cgl*AraE, *Corynebacterium glutamicum* AraE BAH60837.1 (Sasaki et al. 2009); *Eco*AraE, *Escherichia coli* AraE WP_000256438.1 (Daruwalla et al. 1981; Hasona et al. 2004); *Eco*FucP, *E. coli* FucP WP_000528603.1 (Bradley et al. 1987); *Eco*GalP, *E. coli* GalP WP_001112301.1 (Henderson et al. 1977; Hernández-Montalvo et al. 2001); *Eco*XylE, *E. coli* XylE WP_001097274.1 (Lam et al. 1980; Sun et al. 2012); *Lbr*XylT, *Lactobacillus brevis* XylT O52733.1 (Chaillou et al. 1998); *Msm*GlcP, *Mycobacterium smegmatis* GlcP WP_011729622.1 (Pimentel-Schmitt et al. 2009); *Pan*FucP, *Pantoea asanatis* FucP WP_013024528.1 (Andreeva et al., 2013); *Pan*GalP, *P. asanatis* GalP WP_014594508.1 (Andreeva et al. 2013); *Pan*XylE, *P. asanatis* XylE WP_014593545.1 (Andreeva et al. 2013); *Rjo*GluP, *Rhodococcus jostii* GluP WP_009475028.1 (Araki et al. 2011); *Sco*GlcP, *Streptomyces coelicolor* GlcP WP_003971990.1 (van Wezel et al. 2005); *Syn*GlcP, *Synechocystis* sp. PCC6803 GlcP WP_010873345.1 (Zhang et al. 1989); *Zmo*Glf, *Zymomonas mobilis* Glf WP_011240287.1 (Weisser et al. 1995). Sugars which have been identified as substrates are indicated as follows (D-isomers unless otherwise indicated): ara, arabinose; fru, fructose; gal, galactose; glc, glucose; man, mannose; xyl, xylose; 2DG, 2-deoxyglucose; 6DG, 6-deoxyglucose; αMG, methyl-α-glucoside. Multiple alignment of protein sequences was performed using Clustal Omega of the European Bioinformatics Institute (http://www.ebi.ac.uk/Tools/msa/clustalo/) and phylogenetic trees were drawn using TreeView (Page 1996).* Scale bar* = 0.1 amino acid substitution per site

The identity of the putative non-PTS glucose transporter has not been revealed. The genome of *C. beijerinckii* includes two genes (*cbe0109* and *cbe4545*) which encode members of the major facilitator superfamily of proton symporters exhibiting 30–35 % amino acid identity to GlcP (glucose permease) proteins from other organisms. However, a phylogenetic tree of pentose and hexose symporters indicates that the clostridial proteins do not cluster with the GlcP homologues (Fig. 2), and *Cbe*0109 in particular is more closely related to pentose transporters. Nevertheless, determination of the relationship between characterised symporter proteins clearly does not allow for unambiguous prediction of substrate spectrum, since permeases positioned in different branches of the tree have overlapping specificity. As already discussed, the Cbe0109 (XylT) symporter, which is most closely related to the XylT transporter of *Lactobacillus brevis*, has been shown to transport xylose (Xiao et al. 2012), but its ability to transport glucose has not been assessed.

## Regulation of sugar uptake in clostridia

**Fig. 3:**
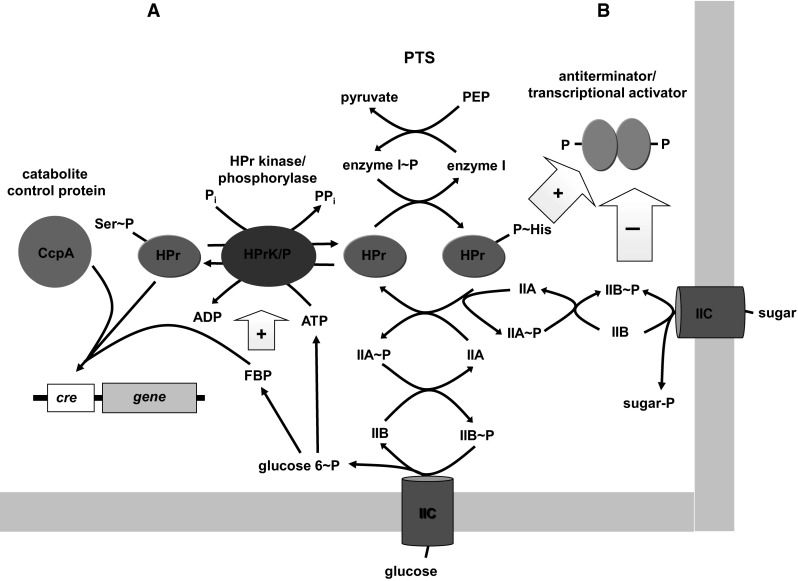
Control of sugar metabolism by glucose in firmicutes.** A** CCR in which CcpA is induced to bind to a target DNA sequence by a seryl-phosphorylated form of HPr (P ~ Ser-HPr) and fructose 1,6-bisphosphate (FBP).** B** Control of *pts* operon expression via PTS-dependent phosphorylation of an antiterminator or transcriptional activator. See text for details

Identification of the functions of individual transport systems can be instrumental in development of engineering strategies to construct clostridial strains that will ferment different substrates effectively. However, a more generally applicable approach is likely to involve targeting of regulatory processes. Regulons associated with uptake and metabolism of xylose and arabinose have been identified in *C. acetobutylicum* and *C. beijerinckii* (Gu et al. 2010; Zhang et al. 2012), and in both organisms increased gene expression following inactivation of a XylR transcriptional repressor has been shown to be associated with increased utilization of xylose as a substrate (Hu et al. 2011; Xiao et al. 2012). Attention has also been directed at manipulating the phenomenon of carbon catabolite repression (CCR) in which a readily metabolised sugar exerts a widespread inhibitory effect on uptake and metabolism of alternative sugar substrates. In firmicutes, glucose exerts control over expression of catabolic operons via two principal mechanisms (Fig. 3). First, a global mechanism of CCR dependent on the catabolite control protein (CcpA) (Warner and Lolkema 2003; Deutscher et al. 2006), which is stimulated to bind to regulatory target sites called catabolite responsive elements (*cre*’s) as a result of interaction with a phosphorylated form of the PTS phosphocarrier protein HPr (P ~ Ser-HPr). Unlike P ~ His-HPr which is generated by PEP-dependent phosphorylation of his 15 catalysed by enzyme I in the course of sugar uptake and phosphorylation, P ~ Ser-HPr is generated by ATP-dependent phosphorylation of ser46, catalysed by a metabolite-activated HPr kinase/phosphorylase (HprK). Stimulation of kinase activity (and DNA binding) by glycolytic intermediates, notably fructose 1,6-bisphosphate, provides the link between glucose metabolism and repression of genes responsible for utilization of a range of substrates. The second mechanism, targeted towards PTS-encoding operons, is mediated by control of activity of PTS-dependent antiterminators and transcriptional activators (Deutscher et al. 2006). Antiterminators comprise an RNA-binding domain and two domains referred to as PRD’s (PTS-regulation domains) which contain conserved histidine residues that are sites for phosphorylation by the PTS. DNA-binding transcriptional activators are larger proteins which, in addition to one or two PRD’s, also include domains resembling the IIA and/or IIB domains of the PTS that provide additional phosphorylatable residues. A number of these regulators have been characterised with respect to their phosphorylation and consequent effects on their activity and expression of the genes that they control (Deutscher et al. 2014). While there is considerable variability between regulators in terms of the domains that are phosphorylated, it has generally been found that phosphorylation by a PTS IIB domain is inhibitory, while phosphorylation by P ~ His-HPr is stimulatory. These influences ensure that operons controlled by the regulators are expressed when required. Thus, uptake of a PTS substrate removes phosphate from the IIB domain of its specific phosphoryl transfer chain, resulting in prevention of phosphorylation of the cognate transcriptional regulator which in turn allows for induction of expression of the associated operon. On the other hand in the presence of glucose, phosphate is diverted from P ~ His-HPr to support glucose uptake with the result that the regulator is inactive and expression of the operon will be repressed (Fig. 3).

Several genes encoding antiterminators and PRD-containing transcriptional activators have been identified in clostridial genomes (Mitchell 2015), but the putative regulatory proteins have not been subjected to experimental scrutiny. Potential ribonucleic antiterminator (RAT) sequences involved in antiterminator binding were identified in *C. acetobutylicum* within the sucrose operon (Tangney and Mitchell 2000) and upstream of the *glcG* gene encoding a glucose PTS (Tangney and Mitchell 2007), but again their role in gene expression has not been verified. On the other hand, *C. acetobutylicum* has been shown to have an active HprK (Tangney et al. 2003), and several studies have focussed on inactivation of the CcpA-dependent pathway of CCR in order to alleviate repression of sugar utilization, in particular effects on metabolism of pentose sugars. Ren et al. (2010) inactivated the *ccpA* gene of *C. acetobutylicum* and demonstrated that the mutant strain exhibited improved utilization of xylose in the presence of glucose, although an undesirable side-effect was the increased accumulation of acetate and butyrate due to decreased expression of the CoA transferase required for assimilation of acids as a first step in solvent formation. A later transcriptional study found that *ccpA* inactivation had a pleiotropic effect on gene expression (both repressive and stimulatory) which extended beyond genes involved in uptake and metabolism of sugars (Ren et al. 2012). *In vitro* analysis of binding of CcpA to representative *cre* sequences validated the role of the protein in control of gene expression; however, it was observed that not all regulated genes were associated with a *cre*, suggesting that in some cases the effect of CcpA was indirect.

A recent analysis has further indicated the scope for precise molecular engineering of the CcpA protein to generate a strain of *C. acetobutylicum* with improved fermentation properties (Wu et al. 2015). Several point mutations were introduced into the protein and screened for effects on xylose fermentation. Of the mutants examined, substitution of val302 within the CcpA regulatory domain was found to have the greatest effect, with replacement by arg (V302N) resulting in optimum xylose utilization as a result of increased expression of xylose degradation pathway genes. However, consistent with the properties of the *ccpA*-disrupted mutant described earlier (Ren et al. 2010), the V302 N mutant also exhibited the undesirable trait of reduced solvent formation, as a result of lowered binding of the mutated protein to relevant target sites. These defects could be remedied by expression of genes associated with solvent formation from a constitutive promoter. Although comparable studies have not been reported for *C. beijerinckii*, its *ccpA* gene has been identified and shown to be capable of complementing a *C. acetobutylicum ccpA* mutant (Gu et al. 2014). It can therefore be anticipated that a similar experimental strategy can be used to improve the fermentation characteristics of this organism.

Attempts have also been made to manipulate CCR in *C. acetobutylicum* by targeting glucose sensing and uptake. Xiao et al. (2011) inactivated the *glcG* gene but, although phosphorylation of the glucose analogue methyl α-glucoside was reduced, extracts of the mutant showed comparable glucose phosphorylation to the parental strain. Nevertheless, repression of utilization of xylose and arabinose in the mutant was partially relieved, suggesting that the GlcG transporter plays some part in the process, while ability to ferment xylose could be further enhanced by the overexpression of genes encoding enzymes of the xylose metabolic pathway. Strain engineering by disruption of glucose uptake is clearly complicated by the fact that the bacterium contains multiple phosphotransferase systems with overlapping substrate specificity, and this is likely to be even more pronounced in *C. beijerinckii* due to the considerably greater number of systems present. It is possible that some systems may be adapted to play a specific sensing or regulatory role, as has been proposed for glucose and β-glucoside phosphotransferases in *Listeria monocytogenes* (Aké et al. 2011; Brehm et al. 1999).

## Conclusion

In terms of detailed molecular analysis, studies on sugar transport in the solventogenic clostridia are less well developed compared to investigations on some other bacteria. Nevertheless, it is clear that the clostridia utilise the same basic mechanisms and control strategies as those found elsewhere. Sugar uptake and its regulation are critical aspects of control of bacterial fermentation, and a thorough characterization can make a significant contribution towards the future development of an effective ABE process. Recent advances in strategies for metabolic engineering of the clostridia have shown the potential for developing strains with improved fermentation characteristics. Based on an understanding of sugar uptake and associated control mechanisms, application of these technologies to optimising sugar utilization should result in key improvements in strain performance.
